# Dual Supramolecular Nanoparticle Vectors Enable CRISPR/Cas9‐Mediated Knockin of Retinoschisin 1 Gene—A Potential Nonviral Therapeutic Solution for X‐Linked Juvenile Retinoschisis

**DOI:** 10.1002/advs.201903432

**Published:** 2020-04-16

**Authors:** Shih‐Jie Chou, Peng Yang, Qian Ban, Yi‐Ping Yang, Mong‐Lien Wang, Chian‐Shiu Chien, Shih‐Jen Chen, Na Sun, Yazhen Zhu, Hongtao Liu, Wenqiao Hui, Tai‐Chi Lin, Fang Wang, Ryan Yue Zhang, Viet Q. Nguyen, Wenfei Liu, Mengxiang Chen, Steve J. Jonas, Paul S. Weiss, Hsian‐Rong Tseng, Shih‐Hwa Chiou

**Affiliations:** ^1^ Division of Basic Research Department of Medical Research and Department of Ophthalmology Taipei Veterans General Hospital Taipei 112 Taiwan; ^2^ Institute of Pharmacology School of Medicine National Yang‐Ming University Taipei 112 Taiwan; ^3^ Department of Molecular and Medical Pharmacology Crump Institute for Molecular Imaging (CIMI) California NanoSystems Institute (CNSI) University of California, Los Angeles Los Angeles CA 90095 USA; ^4^ Center for Stem Cell and Translational Medicine School of Life Sciences Anhui University Hefei 230601 China; ^5^ Department of Medical Research Taipei Veterans General Hospital Taipei 112 Taiwan; ^6^ School of Medicine, and School of Pharmaceutical Sciences National Yang‐Ming University Taipei 112 Taiwan; ^7^ Institute of Food Safety and Health Risk Assessment National Yang Ming University Taipei 112 Taiwan; ^8^ Shandong Provincial Qianfoshan Hospital the First Hospital Affiliated to Shandong First Medical University Jinan 250014 China; ^9^ Institute of Animal Husbandry and Veterinary Medicine Anhui Academy of Agriculture Sciences Hefei 230031 China; ^10^ State Key Laboratory of Molecular Engineering of Polymers Department of Macromolecular Science Fudan University Shanghai 200433 China; ^11^ Department of Chemistry and Biochemistry Department of Bioengineering Department of Materials Science and Engineering California NanoSystems Institute (CNSI) University of California, Los Angeles Los Angeles CA 90095 USA; ^12^ California NanoSystems Institute (CNSI) Department of Pediatrics David Geffen School of Medicine Eli & Edythe Broad Center of Regenerative Medicine and Stem Cell Research Children's Discovery and Innovation Institute University of California, Los Angeles Los Angeles CA 90095 USA

**Keywords:** codelivery, CRISPR/Cas9, gene therapy, retina, supramolecular nanoparticles, X‐linked juvenile retinoschisis

## Abstract

The homology‐independent targeted integration (HITI) strategy enables effective CRISPR/Cas9‐mediated knockin of therapeutic genes in nondividing cells in vivo, promising general therapeutic solutions for treating genetic diseases like X‐linked juvenile retinoschisis. Herein, supramolecular nanoparticle (SMNP) vectors are used for codelivery of two DNA plasmids—CRISPR‐Cas9 genome‐editing system and a therapeutic gene, Retinoschisin 1 (RS1)—enabling clustered regularly interspaced short palindromic repeats (CRISPR)‐associated protein 9 (CRISPR/Cas9) knockin of the RS1 gene with HITI. Through small‐scale combinatorial screenings, two SMNP vectors, with Cas9 and single guide RNA (sgRNA)‐plasmid in one and Donor‐RS1 and green fluorescent protein (GFP)‐plasmid in the other, with optimal delivery performances are identified. These SMNP vectors are then employed for CRISPR/Cas9 knockin of RS1/GFP genes into the mouse Rosa26 safe‐harbor site in vitro and in vivo. The in vivo study involves intravitreally injecting the two SMNP vectors into the mouse eyes, followed by repeated ocular imaging by fundus camera and optical coherence tomography, and pathological and molecular analyses of the harvested retina tissues. Mice ocular organs retain their anatomical integrity, a single‐copy 3.0‐kb RS1/GFP gene is precisely integrated into the Rosa26 site in the retinas, and the integrated RS1/GFP gene is expressed in the retinas, demonstrating CRISPR/Cas9 knockin of RS1/GFP gene.

## Graphical abstract

Two supramolecular nanoparticle (SMNP) vectors codeliver two DNA plasmids—a CRISPR‐Cas9 construct and a therapeutic gene, RS1. Both SMNP vectors are intravitreally injected into the eyes of mice to enable CRISPR/Cas9 knockin of the RS1 gene using the homology‐independent targeted integration (HITI) strategy, to test a potential nonviral therapeutic solution for X‐linked juvenile retinoschisis (XLRS).

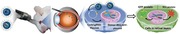

The clustered regularly interspaced short palindromic repeats, CRISPR‐associated protein 9 (CRISPR/Cas9) system is revolutionizing gene therapy.^[^ ^1^ ^]^ A CRISPR/Cas9‐mediated gene editing system is composed of two functional components, i.e., Cas9 endonuclease and an engineered short, single‐guide RNA (sgRNA), which form a ribonucleoprotein complex, Cas9•sgRNA. Based on a base‐pairing mechanism, Cas9•sgRNA complex recognizes and cuts the targeted site, precisely inducing a double‐strand break (DSB).^[^ ^1^ ^]^ Subsequently, endogenous DNA repair occurs via either a nonhomologous end joining (NHEJ) or a homology directed repair (HDR) pathway. As a general therapeutic solution for treating genetic diseases,^[^ ^2^ ^]^ the HDR pathway is often adopted for CRISPR/Cas9‐mediated knockin,^[^ ^3^ ^]^ by which a therapeutic gene carried by donor DNA (dDNA) is integrated into DSB. However, HDR‐based CRISPR/Cas9‐mediated knockin is less efficient in vivo since the HDR pathway is not readily accessible to nondividing cells in tissue.^[^ ^4^ ^]^ By contrast, the NHEJ pathway is active in both nondividing and proliferating cells, and thus, can be adopted to achieve more efficient in vivo CRISPR/Cas9‐mediated knockin. A homology‐independent targeted integration (HITI) strategy^[^ ^5^ ^]^ was developed based on the NHEJ pathway to enable robust knockin of nondividing cells in vivo. In brief, HITI strategy introduces two predetermined CRISPR/Cas9 target sites into dDNA. After cutting the targeted sites present in both genomic DNA and dDNA, the resulting three DSB sites undergoes endogenous DNA repair via the NHEJ pathway to achieve gene integration.^[^ ^5^ ^]^ In animal studies, the HITI strategy enables more efficient CRISPR/Cas9‐mediated knockin, suitable for a local gene‐editing solution in organs, like eyes and brains.^[^ ^5a^ ^]^ However, the technical challenge remains to develop safe and reliable delivery vectors that can codeliver Cas9•sgRNA and dDNA into the target cells and/or tissues.

X‐linked juvenile retinoschisis, XLRS is a condition characterized by impaired vision that begins in childhood in males. Approximately 200 mutations of the RS1 gene have been identified as associated with either decreases in or complete loss of functional retinoschisin, which disrupts the maintenance and organization of cells in the retina.^[^ ^6^ ^]^ Recently, a small number of clinical trials^[^ ^7^ ^]^ were initiated to evaluate the safety and efficacy of adeno‐associated virus (AAV)‐based gene therapy approaches, which introduce functional retinoschisin in retina to treat XLRS. To date, none of the AAV‐based RS1 gene therapy approaches^[^ ^8^ ^]^ has reached a satisfactory clinical endpoint. Meanwhile, researchers have been exploring CRISPR/Cas9‐mediated knockin of RS1 gene to achieve a curative therapeutic solution for XLRS. While AAV^[^ ^9^ ^]^ is also frequently used for delivering CRISPR/Cas9 system, its limited packaging capacity (<4.7 kb)^[^ ^10^ ^]^ and safety concerns on viral integration and immunogenicity remain. Alternative approaches harness the potential of nonviral vectors,^[^ ^11^ ^]^ including lipids,^[^ ^12^ ^]^ polymers,^[^ ^13^ ^]^ and nanoparticles^[^ ^14^ ^]^ for carrying Cas9, guide RNA (gRNA), and donor DNA (dDNA). Although there has been substantial progress made for CRISPR/Cas9‐mediated gene knockout,^[^ ^14^, ^15^ ^]^ and knockdown^[^ ^16^ ^]^ using nonviral vectors along the NHEJ pathway, relatively limited results have been obtained for CRISPR/Cas9‐mediated knockin,^[^ ^14^, ^17^ ^]^ especially for integration of a full‐length gene. We envision exploiting the combined use of nonviral vectors with the more effective HITI strategy to facilitate CRISPR/Cas9‐mediated knockin of a full‐length therapeutic gene as a more effective and general nonviral therapeutic solution for many genetic diseases.

Previously, we demonstrated a convenient and flexible self‐assembled synthetic strategy for producing supramolecular nanoparticle^[^ ^18^ ^]^ (SMNP) vectors by mixing three molecular building blocks, i.e., *β*‐cyclodextrin (CD)‐grafted branched polyethyleneimine (CD‐PEI), adamantane (Ad)‐grafted polyamidoamine dendrimer (Ad‐PAMAM), and Ad‐grafted poly(ethylene glycol) (Ad‐PEG). The multivalent Ad/CD molecular recognition allows modular control over the sizes, surface chemistry, and payloads of SMNP vectors, promising a diversity of imaging^[^ ^18^, ^19^ ^]^ and therapeutic applications.^[^ ^20^ ^]^ We have shown that this self‐assembly strategy can be utilized for combinatorial formulation and screening of SMNPs to optimize formulations with significantly improved delivery performance.^[^ ^21^ ^]^

Our past experience^[^ ^22^ ^]^ in utilizing SMNP vectors for delivery of DNA plasmid prompted us to exploit the combined use of SMNP vectors with the HITI strategy for in vivo CRISPR/Cas9‐mediated knockin of RS1 gene (**Scheme** **1**a) as a potential nonviral therapeutic solution for XLRS. By conducting small‐scale combinatorial formulations and screenings,^[^ ^21^ ^]^ two SMNP vectors, i.e., Cas9/sgRNA‐plasmid⊂SMNPs and Donor‐RS1/GFP‐plasmid⊂SMNPs with optimal delivery performance were identified. We envision that, by performing intravitreal injection of the two SMNP vectors in a mouse model, Cas9/sgRNA‐plasmid (9.3 kb, detailed gene map see Figure S1 in the Supporting Information) and Donor‐RS1/GFP‐plasmid (4.9 kb, detailed gene map see Figure S1 in the Supporting Information) can be introduced into the retina, initiating CRISPR/Cas9‐mediated knockin of RS1/GFP gene in two consecutive steps. In Step 1, Cas9•sgRNA specifically recognizes and cuts a sgRNA (sgRNA‐Rosa26)‐targeted sequence in a mouse Rosa26 safe‐harbor site ^[^ ^23^ ^]^ and two flanked sites adjacent to RS1/GFP genes (within the Donor‐RS1/GFP‐plasmid), resulting in the formation of three DSBs. In Step 2, DNA repair via the NHEJ pathway leads to site‐specific integration of RS1/GFP genes. To prepare for the in vitro study, the B16 mouse melanoma cell line (no RS1 gene expression) was employed as a model system for optimization. Our self‐assembly strategy^[^ ^18^ ^]^ enables precise control over two synthetic variables—(i) Ad‐PAMAM/CD‐PEI ratios, and (ii) the coverage of a membrane penetration ligand, TAT.^[^ ^20c^ ^]^ Through small‐scale combinatorial screenings, optimal performances were identified for the formulations of Cas9/sgRNA‐plasmid⊂SMNPs and Donor‐RS1/GFP‐plasmid⊂SMNPs according to their performances for inducing CRISPR/Cas9‐mediated insertion and deletion events (indels) and GFP transfection, respectively. The two optimized SMNP vectors were then employed to achieve CRISPR/Cas9‐mediated knockin of RS1/GFP gene in growth‐synchronized B16 cells. The resulting RS1/GFP‐knockin B16 cells were further characterized by fluorescence microscopy, polymerase chain reaction (PCR) assay, Sanger sequencing, and quantitative PCR assay to confirm the successful integration of 3.0‐kb RS1/GFP gene. Finally, we explored the feasibility of performing CRISPR/Cas9‐mediated knockin of RS1/GFP gene in mouse retina by intravitreally coinjecting the two SMNP into mice eyes. Ocular imaging with a fundus camera and optical coherence tomography (OCT), as well as pathological and molecular analyses of the harvested retina tissues, were employed to test the success and efficiency CRISPR/Cas9‐mediated knockin of RS1/GFP gene in mouse retinas.

**Scheme 1:**
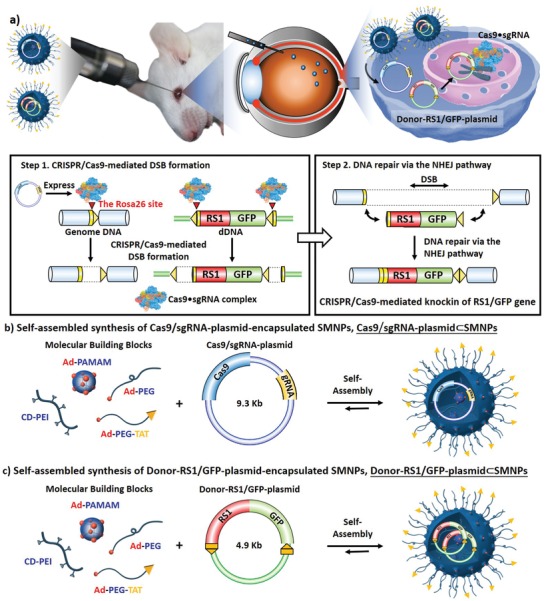
a) Schematic illustration showing that two supramolecular nanoparticle (SMNP) vectors were developed for codelivery of Cas9/sgRNA‐plasmid and Donor‐RS1/GFP‐plasmid, enabling CRISPR/Cas9‐mediated knockin of RS1 gene in mouse retinas. After Cas9•sgRNA formation in vivo, CRISPR/Cas9‐mediated knockin of RS1 gene is carried out in two consecutive steps following the homology‐independent targeted integration (HITI) strategy. b) A self‐assembled synthetic strategy adopted for preparation of Cas9/sgRNA‐plasmid⊂SMNPs through stoichiometric mixing of Cas9/sgRNA‐plasmid and four SMNP molecular building blocks, i.e., CD‐PEI, Ad‐PAMAM, Ad‐PEG, and Ad‐PEG‐TAT. c) A self‐assembly strategy adopted for preparation of Donor‐RS1/GFP‐plasmid⊂SMNPs.

Based on the self‐assembly strategy, we first adopted a small‐scale combinatorial screening approach^[^ ^21^ ^]^ in search of a Cas9/sgRNA‐plasmid⊂SMNPs formulation that optimized performance for CRISPR/Cas9‐mediated disruption at the Rosa26 site (**Figure** **1**a). After cell uptake of Cas9/sgRNA‐plasmid⊂SMNPs, Cas9•sgRNA was produced in cytoplasm, precisely introducing DSB at the Rosa26 site. The subsequent DNA repair via the NHEJ pathway led to the indels. For optimization, 18 formulations of Cas9/sgRNA‐plasmid⊂SMNPs were prepared by systemically varying: (i) Ad‐PAMAM/CD‐PEI weight ratios = 0.5 to 3.0, and (ii) TAT ligand coverage = 1–10%, while keeping the concentrations of Cas9/sgRNA‐plasmid, Ad‐PEG, and CD‐PEI at 0.01, 0.23, and 0.1 µg µL^−1^, respectively. Each formulation of Cas9/sgRNA‐plasmid⊂SMNPs (containing 1.0 µg of Cas9/sgRNA‐plasmid) was introduced to a well (in a 12‐well plate), where 1 × 10^5^ B16 cells were starved in serum‐free Dulbecco's modified Eagle's medium (DMEM) overnight to synchronize the cell cycles to G0/G1 phases.^[^ ^24^ ^]^ 48 h after SMNPs treatment, the B16 cells were subjected for genomic DNA extraction, and the sgRNA‐targeted surrounding region was amplified by PCR. T7 endonuclease I (T7E1) assay^[^ ^3b^ ^]^ (Figure 1b) was performed to quantify the indel frequency. T7 endonuclease specifically recognizes and cleaves mismatched DNA amplicons associated with the indels. Along with the wild‐type (WT) amplicon (574 bp), two characteristic fragments (330 and 244 bp) were detected and quantified by electrophoretogram (Figure 1c), reflecting the CRISPR/Cas9‐mediated disruption performances of the 18 formulations. The optimal performance was identified for a Cas9/sgRNA‐plasmid⊂SMNP formulation, where Ad‐PAMAM/CD‐PEI is 1.5, and TAT coverage is 6%. Replicated CRISPR/Cas9‐mediated disruption tests were performed to ensure the reproducibility of this optimal formulation before knockin study (Figure S2, Supporting Information). The control study, i.e., B16 cells treated by Cas9/sgRNA‐plasmid‐encapsulated Lipofectamine 3000 agent (Cas9/sgRNA‐plasmid⊂LF3K) was conducted in parallel. T7E1 assay revealed that the mean indel frequencies were 23.7% for the Cas9/sgRNA‐plasmid⊂SMNPs and 15.8% for Cas9/sgRNA‐plasmid⊂LF3K (Figure S2, Supporting Information). In addition, to determine potential off‐target effects of Cas9/sgRNA‐plasmid, we performed PCR and Sanger sequence at five of the top‐ranking predicted off‐target sites of the sgRNA‐Rosa26. The sequencing results showed no datable off‐target events in the top five predicted off‐target sites (Figure S3, Supporting Information).

**Figure 1:**
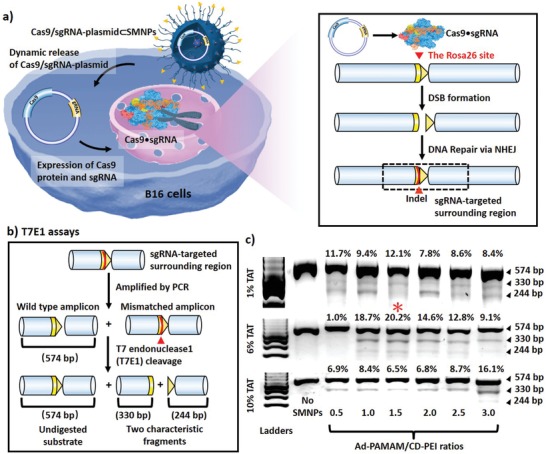
a) Schematic illustration of CRISPR/Cas9‐mediated disruption at the Rosa26 site in B16 cells treated by Cas9/sgRNA‐plasmid⊂SMNPs. After cell uptake of SMNPs, Cas9•sgRNA was produced to introduce DSB precisely at the Rosa26 site. Subsequent DNA repair via the NHEJ pathway led to insertion and deletion events (indels). b) T7 endonuclease I (T7E1) assay was employed to quantify the indel frequency, reflecting the CRISPR/Cas9‐mediated disruption performance. c) Electrophoretograms were used to quantify the two characteristic fragments (330 and 244 bp) associated with the indels along with the wild‐type amplicon (574 bp). An optimal formulation of Cas9/sgRNA‐plasmid⊂SMNPs was identified (*).

Based on a similar combinatorial screening approach,^[^ ^21^ ^]^ we next searched for a Donor‐RS1/GFP‐plasmid⊂SMNPs formulation that optimizes GFP‐transfection performance (**Figure** **2**a). Eighteen formulations of Donor‐RS1/GFP‐plasmid⊂SMNPs were prepared by systemically varying (i) Ad‐PAMAM/CD‐PEI weight ratios = 0.5–3.0, (ii) TAT ligand coverage = 1–10%, while keeping the concentrations of Donor‐RS1/GFP‐plasmid, Ad‐PEG, and CD‐PEI at 0.01, 0.23, and 0.1 µg µL^−1^, respectively. After settling growth‐synchronized B16 cells in culture plates, each formulation of the SMNPs (containing 1.0 µg of Donor‐RS1/GFP‐plasmid) was added to the cells. 48 h post SMNP treatment, fluorescence microscopy was used to quantify the GFP expression levels for individual formulations (Figure 2b, enlarged fluorescence microscopy images see Figure S4 in the Supporting Information). The quantitative analysis summarized in Figure 2c, revealed that the optimal GFP‐transfection performance was identified for a Donor‐RS1/GFP‐plasmid⊂SMNPs formulation, where Ad‐PAMAM/CD‐PEI is 2.0, and TAT coverage is 6%. We further replicated the GFP‐transfection studies of the Donor‐RS1/GFP‐plasmid⊂SMNPs with optimal formulation for three times (Figure S5, Supporting Information). The control study, i.e., B16 cells treated by Donor‐RS1/GFP‐plasmid‐encapsulated Lipofectamine 3000 agent (Donor‐RS1/GFP‐plasmid⊂LF3K), was conducted in parallel. The flow‐cytometry analysis showed the mean transfection efficiencies were 82.9% for Donor‐RS1/GFP‐plasmid⊂SMNPs and 82.5% for Donor‐RS1/GFP‐plasmid⊂LF3K (Figure S5, Supporting Information). Viability of B16 cells after treatment with different SMNP vectors and LF3K vectors was measured via cell counting kit‐8 (CCK‐8) assay. The experimental data (Figure S6, Supporting Information) showed B16 cells kept high viability after treatment of SMNP vectors for 24 and 48 h, while LF3K vectors exhibited a remarkable cytotoxicity against B16 cells.

**Figure 2:**
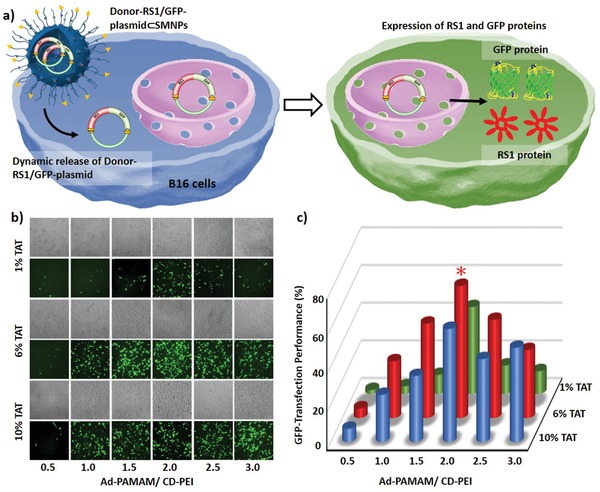
a) Schematic illustration of green fluorescent protein (GFP) transfection in B16 cells treated by Donor‐RS1/GFP‐plasmid⊂SMNPs. b) Eighteen formulations of Donor‐RS1/GFP‐plasmid⊂SMNPs were prepared for the GFP‐transfection study, followed by fluorescence microscopy analysis. c) Quantitative analysis of the fluorescent micrographs revealed an optimal formulation (*) for Donor‐RS1/GFP‐plasmid⊂SMNPs.

The studies above prompted us to explore the feasibility of coencapsulating both Cas9/sgRNA‐plasmid and Donor‐RS1/GFP‐plasmid into a single SMNP vector in order to simplify the complicated procedures using the two vectors. Based on the previous formulation conditions, we prepared Cas9/sgRNA‐plasmid+Donor‐RS1/GFP‐plasmid⊂SMNPs via stoichiometric mixing of the two DNA plasmids with the SMNP building blocks. The resulting Cas9/sgRNA‐plasmid+Donor‐RS1/GFP‐plasmid⊂SMNPs were subjected to both CRISPR/Cas9‐mediated disruption and GFP‐transfection studies. The results (Figure S7, Supporting Information) suggested that such coencapsulated SMNP vector exhibited significantly compromised performance in both CRISPR/Cas9‐mediated disruption (5.1%) and GFP transfection (20%).

Scanning electron microscopy (SEM), transmission electron microscopy (TEM), and dynamic light scattering (DLS) analyses were used to characterize the optimized Cas9/sgRNA‐plasmid⊂SMNPs, Donor‐RS1/GFP‐plasmid⊂SMNPs, and Cas9/sgRNA‐plasmid+Donor‐RS1/GFP‐plasmid⊂SMNPs. The SEM and TEM images and size distributions are shown in **Figure** **3**a,b, and suggest that these two optimized formulations conferred homogeneous spherical morphologies with narrow size distributions to these two SMNP vectors. The coencapsulated SMNPs have larger sizes and size distributions (Figure 3a,b), which might be responsible for their reduced performance. We note that the sizes observed by SEM and TEM are smaller than the hydrodynamic size (Figure S8, Supporting Information), measured by DLS, due to dehydration of SMNPs during sample preparation for electron microscopy. According to stoichiometric calculations (see the Supporting Information), we estimate that ≈1–2 copies of Cas9/sgRNA‐plasmid and 2–3 copies of Donor‐RS1/GFP‐plasmid were encapsulated into each Cas9/sgRNA‐plasmid⊂SMNP and Donor‐RS1/GFP‐plasmid⊂SMNP under the optimized formulation conditions, respectively.

**Figure 3:**
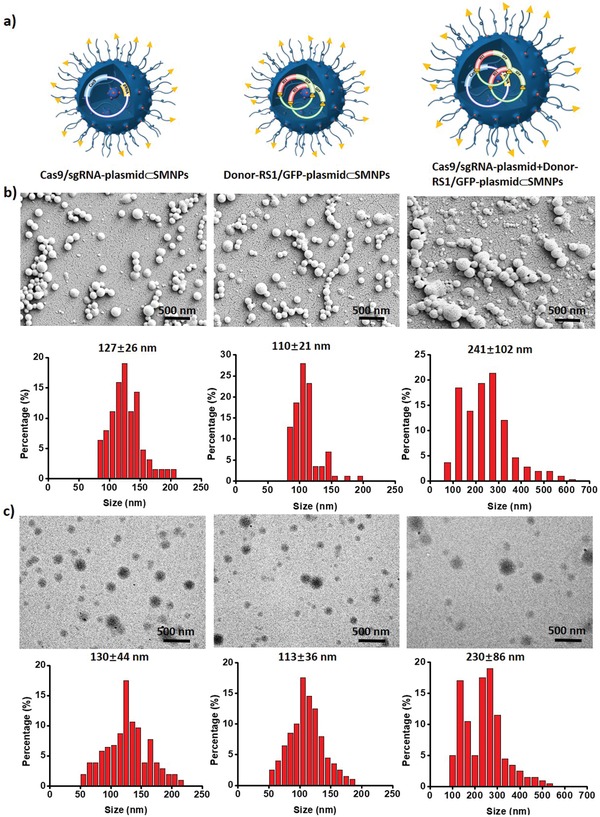
a) Schematic illustrations of optimal Cas9/sgRNA‐plasmid⊂SMNPs, Donor‐RS1/GFP‐plasmid⊂SMNPs, and the coencapsulated Cas9/sgRNA‐plasmid+Donor‐RS1/GFP‐plasmid⊂SMNPs. b) Scanning electron microscopy (SEM) and c) transmission electron microscopy (TEM) images summarize the size distributions of these three SMNPs. The size distributions of SMNPs in the TEM and SEM images were measured via Nano measure 1.2 software. More than 200 SMNPs were counted in each sample.

Using the two optimal SMNPs formulations, growth‐synchronized B16 cells were treated by both Cas9/sgRNA‐plasmid⊂SMNPs and Donor‐RS1/GFP‐plasmid⊂SMNPs (each containing 1.0 µg of plasmid) for CRISPR‐Cas9‐mediated knockin of RS1/GFP gene into the Rosa26 site via the HITI pathway (**Figure** **4**a). To minimize the possibility of positive GFP signals transfected from nonintegrating donor plasmid, a time‐dependent study was conducted on B16 cells treated by both plasmid⊂SMNPs or Donor‐RS1/GFP‐plasmid⊂SMNPs alone at 2, 3, 5, 7, 14, and 21 d post treatment. As shown in Figure S9a in the Supporting Information, the cells treated by Donor‐RS1/GFP‐plasmid⊂SMNPs alone showed the highest GFP‐transfection performance at 3 d post treatment, and the GFP signals gradually decayed and diminished completely by 14 d. The cells treated by both plasmid⊂SMNPs exhibited stable GFP signals post 21 d treatment. After 21 d, the cells were subjected to flow cytometry analysis (Figure S9b,c, Supporting Information) to determine the knockin efficiency (10.7%) and obtain sorted RS1/GFP‐knockin B16 cells. The sorted RS1/GFP‐knockin B16 cells were subjected to 20 rounds of culture expansion. Over the culture expansion, these cells still presented stable and consistent GFP signals (Figure 4b; Figure S10, Supporting Information), supporting the integration of the RS1/GFP gene. To test the success of CRISPR‐Cas9‐mediated knockin of RS1/GFP gene into the Rosa26 site via the HITI pathway, we extracted the genomic DNA from RS1/GFP‐knockin B16 cells, followed by PCR analysis and Sanger sequencing. After PCR amplification, the two characteristic DNA fragments, i.e., the R‐arm junction (617 bp) and L‐arm junction (748 bp)—signifying the integration of 3.0‐kb RS1/GFP into the Rosa26 site—were detected by electrophoresis (Figure 4c). In parallel, Sanger sequencing of the genome‐donor boundaries in the R‐arm and L‐arm junctions confirmed the successful integration of RS1/GFP gene via the HITI pathway (Figure 4d). Moreover, to examine whether the RS1/GFP‐knockin B16 cells were capable of functionally expressing RS1 gene, we carried out quantitative PCR analysis in RS1/GFP‐knockin B16 cells, using untreated B16 cells as controls. We observed a significantly high level of RS1 expression in RS1/GFP‐knockin B16 cells (Figure 4e). RS1/GFP‐knockin B16 cells were subjected to immunofluorescence (IF) staining to examine whether the integrated RS1 gene in RS1/GFP‐knockin B16 cells could functionally express RS1 protein. As shown in Figures 4f and Figure S11 (Supporting Information), strong red fluorescence signals (marking IF‐stained RS1 protein) were observed in RS1/GFP‐knockin B16 cells. Collectively, our in vitro study suggested that the combined use of Cas9/sgRNA‐plasmid⊂SMNPs and Donor‐RS1/GFP‐plasmid⊂SMNPs can successfully achieve CRISPR/Cas9‐mediated knockin of a single‐copy 3.0‐kb RS1/GFP gene into the mouse Rosa26 site. These results represent the first demonstration of CRISPR/Cas9‐mediated knockin of a long/intact gene using nonviral vector‐based gene‐delivery approach.

**Figure 4:**
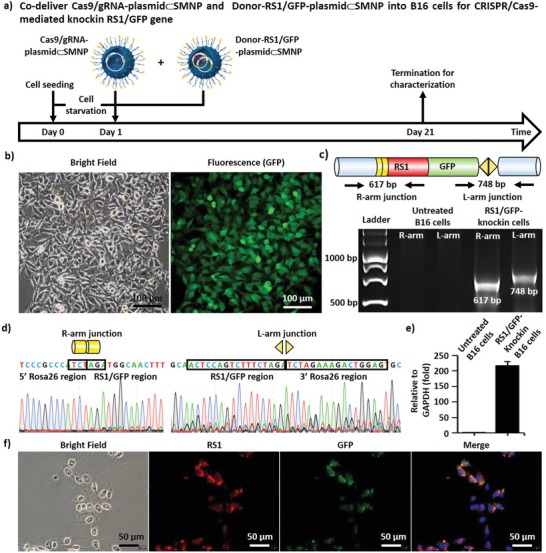
a) A timeline depicting CRISPR/Cas9‐mediated knockin of RS1/GFP gene in growth‐synchronized B16 cells using both Cas9/sgRNA‐plasmid⊂SMNPs and Donor‐RS1/GFP‐plasmid⊂SMNPs. b) Bright‐field and fluorescence images of sorted RS1/GFP‐knockin B16 cells taken after 20 rounds of culture expansion. c) Two characteristic DNA fragments, i.e., the R‐arm junction (617 bp) and L‐arm junction (748 bp)—signifying the integration of RS1/GFP into the Rosa26 site—were detected by an electrophoretogram. d) Sanger sequencing was carried to test that the correct DNA sequences of the genome‐donor boundaries in the R‐arm and L‐arm junctions. e) RS1 gene expression levels observed by quantitative PCR. f) Representative immunofluorescence images of RS1/GFP‐knockin B16 cells.

Following the successful demonstration of in vitro CRISPR/Cas9‐mediated knockin, we were keen to explore the feasibility of performing CRISPR/Cas9 knockin of RS1/GFP gene in vivo. A 5 µL phosphate‐buffered saline (PBS) solution containing sterilized Cas9/sgRNA‐plasmid⊂SMNPs and Donor‐RS1/GFP‐plasmid⊂SMNPs (each 50‐ng plasmid) was injected intravitreally into the eye of a BALB/c‐strain mouse (*n* = 6) under brief isoflurane anesthesia. **Figure** **5**a shows the timeline of our in vivo study over a period of 30 d. At days 3, 10, and 30 postinjection, a fundus camera and OCT were used to measure the knockin GFP signals on retinal surfaces (left and middle panels in Figure 5b and Figure S12a in the Supporting Information) and to monitor the anatomical structures of the retinas (right panels in Figure 5b and Figure S12a in the Supporting Information), respectively. Bright‐field fundus and OCT imaging suggested that the mice retinas retained anatomical integrity over the course of the study. The GFP signals associated with CRISPR/Cas9‐mediated knockin of RS1/GFP gene emerged at day 18 and persisted until day 30. At day 30, the mice were euthanized by cervical dislocation under deep anesthesia, and the treated eyes were excised for pathological and molecular analyses. After dissecting retinas from the treated eyes, the GFP‐positive areas were subjected to standard pathology H&E staining and IHC staining for GFP (Figure 5c; Figure S12b, Supporting Information). Two pathologists reviewed all the slides independently, concluding that: (1) no histological abnormalities were observed, and (2) GFP positivity was identified in the ganglion cell layers of the retinas. In parallel, genomic DNA was extracted from the GFP‐positive retina tissues to confirm that the RS1/GFP gene was correctly integrated into the Rosa26 site. After PCR amplification, the two characteristic fragments, i.e., the R‐arm junction (617 bp) and L‐arm junction (748 bp) were observed on an electrophoretogram (Figure 5d). Sanger sequencing of the genome‐donor boundaries in the R‐arm and L‐arm junctions indicated the successful integration of RS1/GFP gene. (Figure 5e). Collectively, these in vivo experimental data support that the SMNP vectors can be utilized to deliver CRISPR/Cas9 gene editing system and dDNA using the HITI strategy, enabling CRISPR/Cas9‐mediated knockin of intact RS1 gene in retina as a potential nonviral therapeutic solution for treating XLRS.

**Figure 5:**
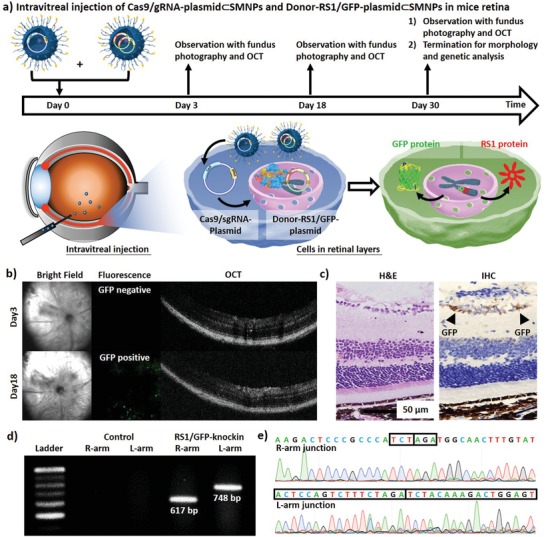
a) A timeline and graphic illustration depicting CRISPR/Cas9‐mediated knockin of the RS1/GFP gene in mouse retina via intravitreal injection of both Cas9/sgRNA‐plasmid⊂SMNPs and Donor‐RS1/GFP‐plasmid⊂SMNPs. b) A fundus camera and optical coherence tomography (OCT) were employed to detect the GFP signals on retinal surfaces and monitor the anatomical structures of the retinas, respectively. c) H&E staining and IHC staining for GFP of the GFP‐positive retina tissues. d) Two characteristic DNA fragments, i.e., the R‐arm junction (617 bp) and L‐arm junction (748 bp) on an electrophoretogram and e) Sanger sequencing of the genome‐donor boundaries in the R‐arm and L‐arm junctions confirmed the successful integration of 3.0‐kb RS1/GFP gene into the Rosa26 site in vivo.

In summary, we introduced an in vivo CRISPR/Cas9‐mediated knockin approach using two SMNP vectors, i.e., Cas9/sgRNA‐plasmid⊂SMNPs and Donor‐RS1/GFP‐plasmid⊂SMNPs, which were identified by performing small‐scale combinatorial screenings of the different SMNP formulations prepared by a self‐assembly synthetic strategy. By intravitreally injecting the two SMNP vectors in BALB/c‐strain mice, we successfully demonstrated CRISPR/Cas9‐mediated knockin of 3.0‐kb RS1/GFP gene into the Rosa26 site in mice retinas. This proof‐of‐concept study highlights the potential of the combined use of the SMNP vectors with the HITI strategy to achieve in vivo CRISPR/Cas9‐mediated knockin of a therapeutic gene. Since nearly 200 mutation sites in the RS1 gene have been identified and associated with XLRS, it is not feasible to develop CRISPR/Cas9‐mediated editing according to individual patients’ mutations. Performing CRISPR/Cas9‐mediated knockin of the RS1 gene in the retinas of XLRS patients via either intravitreal or subretinal injection of the SMNPs would offer a revolutionary curative therapeutic solution. The same strategy should apply for treating other genetic diseases in which specific mutations only function in local organs (e.g., cystic fibrosis).

## Conflict of Interest

The authors declare no conflict of interest.

## Supporting information

Supporting Information
