# Supplementary material for: Dual Supramolecular Nanoparticle Vectors Enable CRISPR/Cas9‐Mediated Knockin of Retinoschisin 1 Gene—A Potential Nonviral Therapeutic Solution for X‐Linked Juvenile Retinoschisis
Source: Adv Sci (Weinh). 2020 Apr 16;7(10):1903432. doi: 10.1002/advs.201903432 (PMC7237855; doi:10.1002/advs.201903432)
Supplement: Supplementary file 1 — Supporting Information [file ADVS-7-1903432-s001.pdf]

Copyright WILEY-VCH Verlag GmbH & Co. KGaA, 69469 Weinheim, Germany, 2020.

Supporting Information

Dual Supramolecular Nanoparticle Vectors Enable CRISPR/Cas9-Mediated Knockin of Retinoschisin 1 Gene – A Potential Non-Viral Therapeutic Solution for X-Linked Juvenile Retinoschisis

Shih-Jie Chou, Peng Yang, Qian Ban, Yi-Ping Yang, Mong-Lien Wang, Chian-Shiu Chien, Shih-Jen Chen, Na Sun, Yazhen Zhu, Hongtao Liu, Wenqiao Hui, Tai-Chi Lin, Fang Wang, Ryan Yue Zhang, Vient Q. Nguyen, Wenfei Liu, Mengxiang Chen, Steve J. Jonas, Paul S. Weiss, Hsian-Rong Tseng,* and Shih-Hwa Chiou**

Dr. S.-J. Chou, Dr. C.-S. Chien, Dr. S.-J. Chen, Dr. T.-C. Lin, V. Nguyen, Prof. S.-H. Chiou
Division of Basic Research, Department of Medical Research, and Department of Ophthalmology, Taipei Veterans General Hospital, Taipei 112, Taiwan. Institute of Pharmacology, School of Medicine, National Yang-Ming University, Taipei, 112, Taiwan
E-mail: shchiou@vghtpe.gov.tw

Dr. P. Yang, Dr. N. Sun, Dr. Y. Zhu, R. Y. Zhang, M. Chen, Prof. H.-R. Tseng
Department of Molecular and Medical Pharmacology, Crump Institute for Molecular Imaging (CIMI), California NanoSystems Institute (CNSI), University of California, Los Angeles, Los Angeles, California 90095, United States
E-mail: hrtseng@mednet.ucla.edu

Dr. Q. Ban
Center for Stem Cell and Translational Medicine, School of Life Sciences, Anhui University, Hefei 230601, China

Dr. Y.-P. Yang
Department of Medical Research, Taipei Veterans General Hospital, Taipei, 112, Taiwan. School of Medicine, and School of Pharmaceutical Sciences, National Yang-Ming University, Taipei, 112, Taiwan

Dr. M.-L. Wang
Department of Medical Research, Taipei Veterans General Hospital, Taipei, 112, Taiwan. Institute of Pharmacology, School of Medicine, National Yang-Ming University, Taipei, 112, Taiwan. Institute of Food Safety and Health Risk Assessment, National Yang Ming University, Taipei, 112, Taiwan

Dr. H. Liu
Shandong Provincial Qianfoshan Hospital, the First Hospital Affiliated to Shandong First Medical University, Jinan, 250014, China

Dr. W. Hui
Institute of Animal Husbandry and Veterinary Medicine, Anhui Academy of Agriculture Sciences, Hefei, 230031, China

Dr. F. Wang
State Key Laboratory of Molecular Engineering of Polymers, Department of Macromolecular Science, Fudan University, Shanghai, 200433, China

Prof. S. J. Jonas
California NanoSystems Institute (CNSI), Department of Pediatrics, David Geffen School of Medicine, Eli & Edythe Broad Center of Regenerative Medicine and Stem Cell Research, Children's Discovery and Innovation Institute, University of California, Los Angeles, Los Angeles, California 90095, United States

W. Liu, Prof. P. S. Weiss
Department of Chemistry and Biochemistry, Department of Bioengineering, Department of Materials Science and Engineering, California NanoSystems Institute (CNSI), University of California, Los Angeles, Los Angeles, California 90095, United States
E-mail: psw@cnsi.ucla.edu

S.-J. Chou and P. Yang contributed equally to this work.

METHODS SECTION

Materials and Methods

Chemical reagents and solvents were purchased from Sigma (Missouri) and were used as received without further purification unless otherwise noted. Cas9/sgRNA-plasmid was a gift from Ralf Kuehn and synthesized from Addgene (Addgene plasmid #64216, http://n2t.net/addgene:64216, RRID: Addgene_64216, Massachusetts) Donor-RS1/GFP-plasmid was synthesized from GeneCopoeia (Maryland). Lipofectamine 3000 agent was purchased from ThermoFisher Scientific (Massachusetts) and the methods for Lipofectamine 3000 followed the protocols on ThermoFisher Scientific website. Cell Counting Kit-8 (CCK8) was purchased from Abcam (Cambridge, United Kingdom) and the protocol can be found on Abcam website. B16 cells (mouse melanoma cell line) were purchased from American Type Culture Collection (ATCC, Virginia) and maintained in Dulbeco's modified Eagle's medium (DMEM, Gibco, Massachusetts) with 10% Fetal bovine serum (FBS, Gibco) and 1% penicillin-streptomycin (Gibco). For Indel events analysis: GeneArt® Genomic Cleavage Detection Kit was purchased from ThermoFisher Scientific. DNA extraction kit was purchased from Qiagen (QIAamp® DNA Mini Kit, Hilden, Germany). All experimental procedures and protocols involving animals were approved by the institutional animal care committee of Taipei Veterans General Hospital and complied with the Guide for the Care and Use of Laboratory Animals.

Measurements

Dynamic light scattering (DLS) was measured on Zetasizer Nano instrument (Malvern Instruments Ltd., United Kingdom) equipped with a 10-mW helium-neon laser (λ = 632.8 nm) and a thermoelectric temperature controller. Measurements were taken at a 90° scattering angle with 0.3% attenuation. The concentration of the SMNPs was 1 mg/mL. Each sample was replicated over 3 runs.

NanoDrop™ 2000/c Spectrophotometers (Thermo Scientific, Massachusetts) was used to test the plasmid concentration in upper supernatant after centrifuge SMNPs to check the loading efficiencies of Cas9/sgRNA-plasmid and Donor-RS1/GFP-plasmid in SMNP vectors.

Cell imaging studies were performed on a Nikon TE2000S inverted fluorescent microscope with a cooled charge-coupled device (CCD) camera (QImaging, Retiga 4000R), X-Cite 120 Mercury lamp, automatic stage, and filters for five fluorescent channels (W1: 325-375 nm, W2: 465-495 nm, W3: 570-590 nm, W4: 590-650 nm, and W5: 650-900 nm). Fluorescence intensities were measured by a Fujifilm BAS-5000 microplate reader.

Scanning electron microscope (SEM) images were performed on a TS-5136MM (TESCAN, Czech) scanning electron microscope at an accelerating voltage of 20 kV. Samples dispersed at an appropriate concentration were cast onto a glass sheet at room temperature and sputter-coated with gold.

Transmission electron microscope (TEM) images were carried out on a Philips CM 120 electron microscope, operating at an acceleration voltage of 120 kV. The TEM samples were prepared by drop-coating 2 μL of sample suspension solutions onto carbon-coated copper grids. Excess amounts of solution were removed by filter papers after 45 s. Subsequently, the samples were negatively stained with 2% uranyl acetate for 45 s before TEM studies.

Cell culture

The mouse melanoma B16 cells were cultured in a humidified atmosphere of 5% CO_2/air in DMEM medium, supplemented with 10% fetal bovine serum, 100 units/mL penicillin and 100 μg/mL streptomycin. Individual wells of a 12-well plate were inoculated with complete medium containing 100,000 of B16 cells per well. The plates were incubated at 37 °C in a humidified 5% incubator for 18 h prior to the experiments.

Synthesis of Cas9/sgRNA-plasmid⊂SMNPs

Self-assembly was used to prepare the Cas9/sgRNA-plasmid encapsulated supramolecular nanoparticles (Cas9/sgRNA-plasmid⊂SMNPs).[1] Eighteen formulations of Cas9/sgRNA-plasmid⊂SMNPs were prepared via systemically modulating i) the weight ratios (0.5 to 3.0) between Ad-PAMAM and CD-PEI, ii) the percentages (1% to 10%) of TAT ligand on SMNP surfaces, while keeping the concentrations of Cas9/sgRNA-plasmid, Ad-PEG, and CD-PEI at 0.01, 0.23, and 0.1 μg/μL, respectively. The optimized synthesis formulation follows. The optimal synthesis formulation is below: A total of 2.0 μL DMSO solution containing Ad-PAMAM (15 μg) was added into a 100 μL PBS mixture with Cas9/sgRNA-plasmid (1.0 μg), Ad-PEG (23 μg), CD-PEI (10 μg), and Ad-PEG-TAT (1.4 μg). The resulting mixture was then stirred vigorously to achieve optimal Cas9/sgRNA-plasmid⊂SMNPs. The mixture was stored at 4 ℃ for 1 h; after that, DLS, SEM and TEM were used to character the sizes of Cas9/sgRNA-plasmid⊂SMNPs. The loading efficiency of Cas9/sgRNA-plasmid in SMNP vectors was 85%, checked by NanoDrop™ 2000/c Spectrophotometers.

Synthesis of Donor-RS1/GFP-plasmid⊂SMNPs

A similar self-assembly procedure was used to prepare the Donor-RS1/GFP-plasmid⊂SMNPs. Eighteen formulations of Donor-RS1/GFP-plasmid⊂SMNPs were prepared via systemically modulating i) the weight ratios (0.5 to 3.0) between Ad-PAMAM and CD-PEI, ii) the percentages (1% to 10%) of TAT ligand on SMNP surfaces, while keeping the concentrations of Donor-RS1/GFP-plasmid, Ad-PEG, and CD-PEI at 0.01, 0.23, and 0.1 μg/μL, respectively. The optimized synthesis formulation follows. The optimal synthesis formulation is below: A total of 2.0 μL DMSO solution containing Ad-PAMAM (20 μg) was added into a 100 μL PBS mixture with Cas9/GFP-plasmid (1.0 μg), Ad-PEG (23 μg), CD-PEI (10 μg), and Ad-PEG-TAT (1.4 μg). The above resulting mixture was then stirred vigorously to achieve optimal Donor-RS1/GFP-plasmid⊂SMNPs. The mixture was stored at 4℃ for 30min, after that, DLS, SEM and TEM were used to characterize the sizes of EGFP-

Cas9•sgRNA⊂SMNPs. The loading efficiency of Donor-RS1/GFP-plasmid in SMNP voctors was 92%, checked by NanoDrop™ 2000/c Spectrophotometers.

Delivery of Cas9/sgRNA-plasmid⊂SMNPs or Donor-RS1/GFP-plasmid⊂SMNPs into B16 cells

Prior to settling the cells onto 12 well plates, B16 cells were starved in serum-free DMEM overnight (18 h) to synchronize cells to G0/G1 phases of cell cycle.[2] B16 cells (1×105) were introduced into each well of a 12-well plate. After starvation, the medium was replaced by 2.0 mL DMEM medium containing 10% fetal bovine serum, and then, different formulations of Cas9/sgRNA-plasmid⊂SMNPs or Donor-RS1/GFP-plasmid⊂SMNPs (containing 1.0 μg of Cas9/sgRNA-plasmid or 1.0 μg of Donor-RS1/GFP-plasmid) were introduced into each well and incubated with cells for the designated time. Every 48 h, 1.0 mL medium was removed via pipette and replaced by 1.0 mL serum-containing DMEM medium in each well. The cells were spitted and seeded in 10-cm dishes upon reaching 80-90% confluence. T7E1 assay was performed to quantify the indel frequencies of different formulations of Cas9/sgRNA-plasmid⊂SMNPs. Microscopy-based image cytometry was used to detect the cellular uptake performances of different formulations Donor-RS1/GFP-plasmid⊂SMNPs. The GFP signal was quantified with fluorescent microscope with a CCD camera (Nikon H550, Japan).

T7E1 assay

The T7E1 assay was performed by using GeneArt™ Genomic Cleavage Detection Kit (purchased from ThermFisher, A24372). In brief, genomic DNA of the transfected cells were extracted by Cell Lysis Buffer. PCR products were purified with AmpliTaq Gold® 360 Master Mix and were denatured and annealed by using S1000TM Thermal Cycler (Bio-Rad). Hybridized PCR products were digested with Detection Enzyme at 37 °C for 1 h and then run through 2% agarose gel electrophoresis.

The PCR primer sequences used were:

Rosa26_T7E1_F: TACTCCGAGGCGGATCACAA

Rosa26_T7E1_R: GCAAGCACGTTTCCGACTTG

Off-target analysis

Off-target analysis of sgRNA-Rosa26 was used as a query for the Cas-OFFinder design tool (http://www.rgenome.net/cas-offinder/).[3] The top 5 off-target hits with canonical PAM sequence were:

AtgCCAGTCaTTCTAGAAGATGG (chromosome 3 minus strand),

ACTCCctTCTTTCTgGAAGATGG (chromosome 7 minus strand),

ACTtCAGaCaTTCTAGAAGAGGG (chromosome 7 plus strand),

ACTgCAGTgTTTCTAGAAaATGG (chromosome 5 plus strand),

ACcCaAtTCTTTCTAGAAGAAGG (chromosome 16 minus strand).

The PCR primers for these regions were designed accordingly.

Synthesis of Cas9/sgRNA-plasmid+Donor-RS1/GFP-plasmid⊂SMNPs

A similar self-assembly procedure was applied to prepare the Donor-RS1/GFP-plasmid⊂SMNPs. The synthesis formulation follows. A total of 4.0 μL DMSO solution containing Ad-PAMAM (35 μg) was added into a 200 μL PBS mixture with Cas9/sgRNA-plasmid (1.0 μg), Donor-RS1/GFP-plasmid (1.0 μg), Ad-PEG (46 μg), CD-PEI (20 μg), and Ad-PEG-TAT (2.8 μg). The above resulting mixture was then stirred vigorously to achieve Cas9/sgRNA-plasmid+Donor-RS1/GFP-plasmid⊂SMNPs. The mixture was stored at 4℃ for 30min, after that, DLS, SEM and TEM were used to character the sizes of SMNPs.

Delivery of Cas9/sgRNA-plasmid+Donor-RS1/GFP-plasmid⊂SMNPs into B16 cells

Prior to settling the cells onto 12 well plates, B16 cells were starved in serum-free DMEM overnight (18 h) to synchronize cells to the G0/G1 phases of cell cycle.[2] B16 cells (1×10^{5}) were introduced into each well of a 12-well plate. The Cas9/sgRNA-plasmid+Donor-RS1/GFP-plasmid ⊂SMNPs (containing 1.0 µg of Cas9/sgRNA-plasmid and 1.0 µg of Donor-RS1/GFP-plasmid) were added to the well. The cells were co-incubated with SMNPs for 48 h. Microscopy-based image cytometry was used to detect the cellular uptake performances of different formulations. After different treatments, the GFP signal was quantified with fluorescent microscope with a CCD camera (Nikon H550, Japan). T7E1 assay was performed to quantify the frequencies of the Indel events.

Stoichiometric calculations for the number of Cas9/sgRNA-plasmid and Donor-RS1/GFP-plasmid encapsulated into each Cas9/sgRNA-plasmid⊂SMNP and Donor-RS1/GFP-plasmid⊂SMNP, respectively

1. The total number of Cas9/sgRNA-plasmid⊂SMNPs, n_{vector} :

$$n_{vector} = \frac{m_{total\,vectors}}{m_{vector}} = \frac{m_{total\,vectors}}{\frac{4}{3}\pi r^3 \rho} \qquad (1)$$

where $m_{total\,vectors}$ is the total mass of Cas9/sgRNA-plasmid⊂SMNPs (50.4 µg), r is the radius of SMNP vector (65 nm), ρ is the density of SMNP vector (1.1 g/cm^3). By calculation, n_{vector}=4×10^{10}.

The total number of Cas9/sgRNA-plasmid, $n_{Cas9/sgRNA-plasmid}$:

$$n_{Cas9/sgRNA-plasmid} = \frac{m_{total\,Cas9/sgRNA-plasmid}}{M_{Cas9/sgRNA-plasmid}} N_A \qquad (2)$$

where $m_{total\ Cas9/sgRNA-plasmid}$ is the total mass of Cas9/sgRNA-plasmid (0.85 μg), $M_{Cas9/sgRNA-plasmid}$ is the molecular weight of Cas9/sgRNA-plasmid (10 kb≈6,600 kDa), N_A is the Avogadro constant (6.02×10^{23}). By calculation, $n_{\mathrm{Cas9/sgRNA-plasmid}}$=7.3×10^{10}.

The number of Cas9/sgRNA-plasmid encapsulated into each Cas9/sgRNA-plasmid⊂SMNP, $n_{Cas9/sgRNA-plasmid/vector}$:

$$n_{Cas9/sgRNA-plasmid/vector} = \frac{n_{Cas9/sgRNA-plasmid}}{n_{vector}} \approx 2 \quad (3)$$

2. The total number of Donor-RS1/GFP-plasmid⊂SMNPs, n_{vector} :

$$n_{vector} = \frac{m_{total\ vectors}}{m_{vector}} = \frac{m_{total\ vectors}}{\frac{4}{3}\pi r^3 \rho} \quad (4)$$

where $m_{total\ vectors}$ is the total mass of Donor-RS1/GFP-plasmid⊂SMNPs (55.4 μg), r is the radius of SMNP vector (55 nm), ρ is the density of SMNP vector (1.1 g/cm^3). By calculation, n_{vector}=7.1×10^{10}.

The total number of Donor-RS1/GFP-plasmid, $n_{Donor-RS1/GFP-plasmid}$:

$$n_{Donor-RS1/GFP-plasmid} = \frac{m_{total\ Donor-RS1/GFP-plasmid}}{M_{Donor-RS1/GFP-plasmid}} N_A \quad (5)$$

where $m_{total\ Donor-RS1/GFP-plasmid}$ is the total mass of Donor-RS1/GFP-plasmid (0.92 μg), $M_{Donor-RS1/GFP-plasmid}$ is the molecular weight of Donor-RS1/GFP-plasmid (5.2 kb ≈ 3,200 kDa), N_A is the Avogadro constant (6.02×10^{23}). By calculation, $n_{Donor-RS1/GFP-plasmid}$=1.7×10^{11}.

The number of Donor-RS1/GFP-plasmid encapsulated into each Donor-RS1/GFP-plasmid⊂SMNP, $n_{Donor-RS1/GFP-plasmid/vector}$:

$$n_{Donor-RS1/GFP-plasmid/vector} = \frac{n_{Donor-RS1/GFP-plasmid}}{n_{vector}} \approx 3 \quad (6)$$

Co-delivery Cas9/sgRNA-plasmid⊂SMNPs and Donor-RS1/GFP-plasmid⊂SMNPs into B16 cells

Prior to the treatment with SNMP vectors (optimal formulations), B16 cells (1×10^5 per well) were settled in 12-well plates. The cells were starved in serum-free DMEM overnight (18 h) to synchronize cells to the G0/G1 phases of cell cycle. After starvation, the medium was replaced by 2.0 mL DMEM medium containing 10% fetal bovine serum, and then, Cas9/sgRNA-plasmid⊂SMNPs and Donor-RS1/GFP-plasmid⊂SMNPs (containing 1.0 μg of Cas9/sgRNA-plasmid and 1.0 μg of Donor-RS1/GFP-plasmid) were introduced into each well and incubated with cells for the designated time. Every 48 h, 1.0 mL medium was removed via pipette and replaced by 1.0 mL serum-containing DMEM medium in each well. The cells were spitted and seeded in 10-cm dishes upon reaching 80-90% confluence. 21 days post treatment, the cells were subjected to flow cytometry analysis to determine the knockin efficiency and obtain purified RS1/GFP-knockin B16 cells.

DNA extraction and PCR

The RS1/GFP-knockin B16 cells were harvested and then washed with PBS. The genomic DNA was extracted with a commercial QIAamp® DNA Mini Kit (Qiagen, Germany), following manufacturer's instructions. Then, PCR was conducted to amplify integrated RS1/GFP gene with a S1000TM Thermal Cycler (Bio-Rad) under the following PCR conditions: 95 °C for 10 min followed by 40 cycles (95 °C for 15 s, 55 °C for 15 s and 72°C for 30 s) and 72 °C for 5 min. The PCR products were checked on a 1.5% electrophoresis gel.

The PCR primer sequences were listed as follow:

L junction_F: ATGCCAATGCTCTGTCTAGGG

L junction_R: TTCTCTAGGCACCGGTTCAAT

R junction_F: CATCATCTCCCGCTTCATCCG

R junction_R: CAAGCACGTTTCCGACTTGA

Quantitative PCR for RS1 gene expression levels

After adding TRIzol (800 µL), the cells were homogenized, treated with chloroform (160 µL) and centrifuged for 15 min at 4 °C. The aqueous phase of the sample was removed by pipet and 100% isopropanol (400 µL) was added. After being centrifuged for 10 min, the supernatant was removed from the tube, and the pellet was washed with 75% ethanol and centrifuged for 5 min. Afterwards, the supernatant was removed, and the pellet was dissolved in DNase- and RNase-free water. RNA (1 µg) was reverse-transcribed using the SuperScript III First-Strand Synthesis kit. qPCR analysis was performed using PowerUp SYBR Green Master Mix (Applied Biosystems) with the primers. Values were normalized against the gene expression of the housekeeping gene Gapdh.

The qPCR primer sequences were listed as follow:

RS1_F_q: GATTGCCAAGGAGGACCCAA

RS1_R_q: GACCTCCCCTGACTCGAAAC

Gapdh_F_q: TGTGAACGGATTTGGCCGTA

Gapdh_R_q: ACTGTGCCGTTGAATTTGCC

Quantitative PCR for copy numbers

Genomic DNA was extracted by using the Blood & Cell Culture DNA Mini Kit (QIAGEN). qPCR was performed with Fast SYBR™ Green Master Mix according to the manufacturer's instructions (ThermoFisher). The signals were detected with a SimpliAmp Thermal Cycler (ThermoFisher) using 10 ng of purified DNA in 20 µL reactions.

The PCR primer sequences used were:

RS1_F_q: GATTGCCAAGGAGGACCCAA

RS1_R_q: GACCTCCCCTGACTCGAAAC

gGapdh_F_q: TGCGACTTCAACAGCAACTCC

gGapdh_R_q: GCTCTTAAAAGTCAGGTTTCCCAT

Immunofluorescence staining

The living cells were fixed in 4% paraformaldehyde, permeabilized in 0.1% Triton X-100, and blocked in 5% normal bovine serum albumin (BSA) in PBS. The cells were incubated with RS1 (1:500; Abcam) and GFP (1:500; Cell Signaling Technology) antibody. After being washed three times with PBS, the cells were incubated with secondary antibodies conjugated with FITC (green) and Cy3 (red). DAPI (blue) was used as the nuclear stain. Labeled cells were imaged with a laser-scanning confocal microscope (Olympus). The total amount of retained immunofluorescent material was determined in the green (488 nm) and the red (546) channels.

Intravitreal injection

C57BL/6 male mice (6~10 weeks old) were purchased from National Laboratory Animal Center (Taipei, Taiwan). The mice were housed in a pathogen-free space and operated according to the National Research Council's Guide for the Care and Use of Laboratory Animals. All anesthesia and sacrifice procedures were reviewed and approved by the Animal Care and Use Committee of the Taipei Veterans General Hospital (TVGH). The mice were anesthetized with 250mg/kg tribromoethanol (Sigma-Aldrich) by intraperitoneal injection, and placed under a dissecting microscope (SZX16, OLYMPUS, Japan) or spectral-domain OCT imaging system. Each mouse was intravitreally injected with 5 μL of optimal Cas9/sgRNA-plasmid⊂SMNPs and Donor-RS1/GFP-plasmid⊂SMNPs into both eyes. A Hamilton syringe was used to inject 5 μL of the vectors into the vitreous cavity of an eye

through the sclera behind the limbus of mice. The OCT images of the mouse retinas were obtained using a continuous, high-speed and high-resolution retinal image acquisition system (axial resolution, 7 μm; acquisition speed, 76 frames/s, 1000 × 1024 pixels in the X-Z plane). A horizontal scan of 400 images was obtained through the fundus.

H&E staining and IHC

Thirty days after injection, the mice eyes were collected and fixed with 4% paraformaldehyde. The paraffin-embedded tissue was sectioned and stained with hematoxylin and eosin (H&E). The paraffin embedded sections were deparaffinized and rehydrated in Target Retrieval Solution (DaKo). Sections were blocked with 3% fetal bovine serum for 5 min and incubated with the primary antibodies for 30 min at room temperature. The primary antibodies used in this assay were anti-GFP (1:100; Cell Signaling).

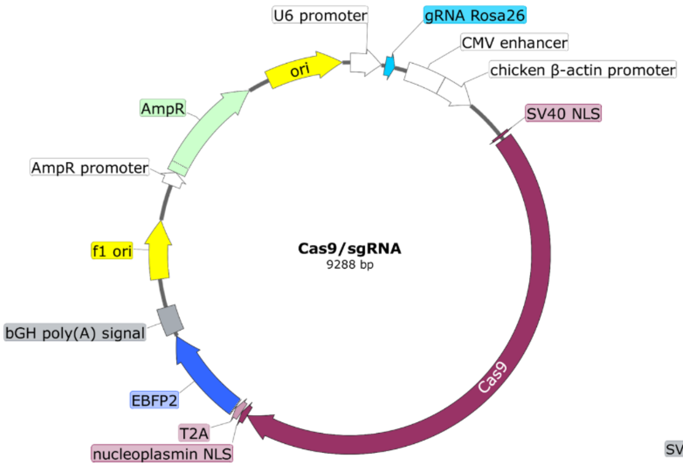

Figure S1. Detailed maps of Cas9/sgRNA-plasmid and Donor-RS1/ GFP-plasmid.

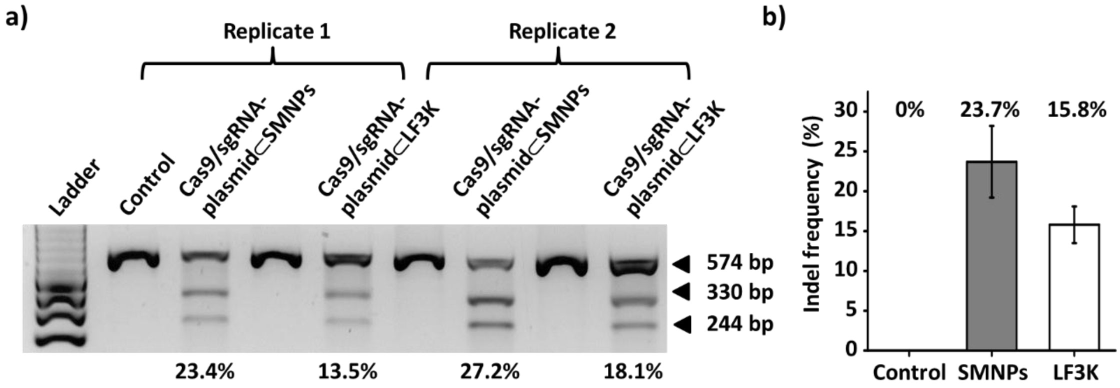

Figure S2. Electrophoretograms were used to quantify the two characteristic fragments (330 bp and 244 bp) associated with the indels along with the wild-type amplicon (574 bp) at 48 h post treatment with Cas9/sgRNA-plasmid⊂SMNP or Cas9/sgRNA-plasmid⊂LF3K. b) Histogram of the indel frequency of B16 cells at 48 h post treatment with Cas9/sgRNA-plasmid⊂SMNP or Cas9/sgRNA-plasmid⊂LF3K.

Top 5 Off-target Loci		
Off-target Locus Location	gRNA sequence (w/ PAM)	PCR Product
Chr3: 62014290 (reverse)	AtgCCAGTCaTTCTAGAAGATGG	524 bp
Chr7: 46223489	ACTCCctTCTTTCTgGAAGATGG	843 bp
Chr7: 65473025	ACTtCAGaCaTTCTAGAAGAGGG	591 bp
Chr5: 99517904	ACTgCAGTgTTTCTAGAAaATGG	975 bp
Chr16: 56589106 (reverse)	ACcCaAtTCTTTCTAGAAGAAGG	607 bp

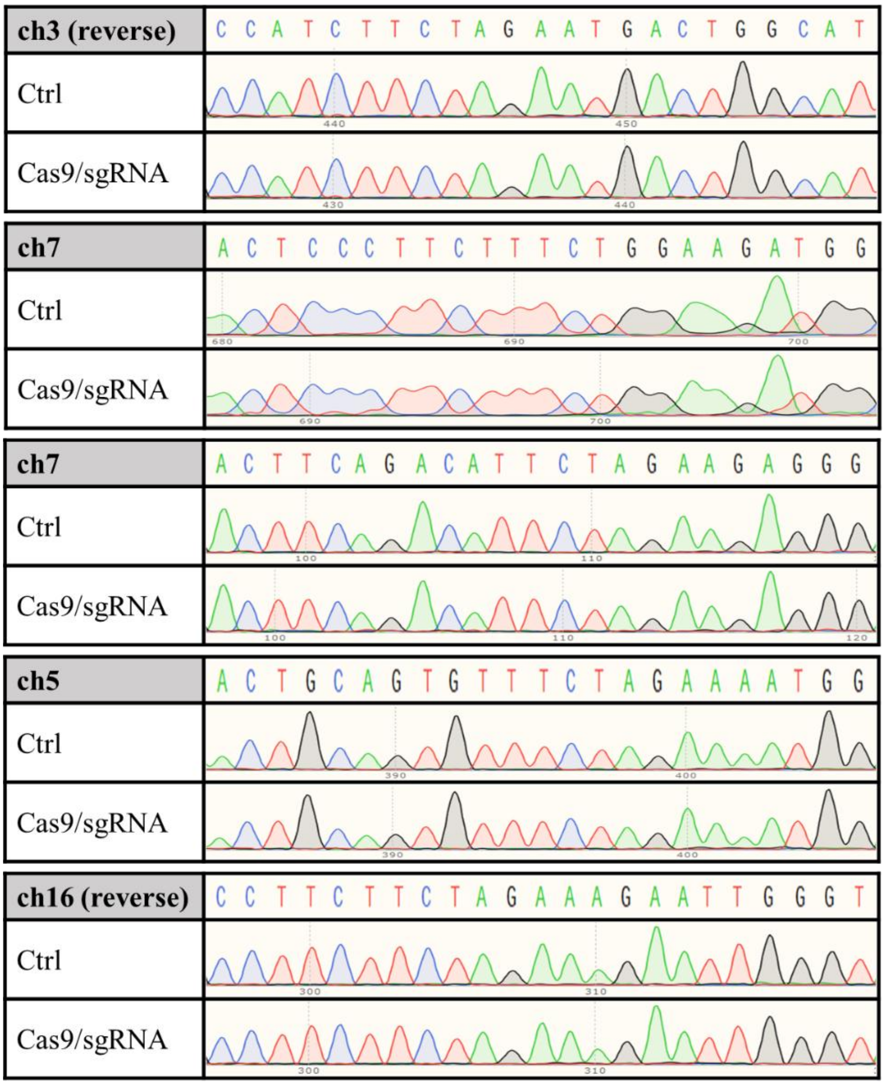

Figure S3. Off-target analysis in B16 cells treated by Cas9/sgRNA-plasmid⊂SMNPs. The sequencing results showed no off-target events in the top 5 predicted off-target sites.

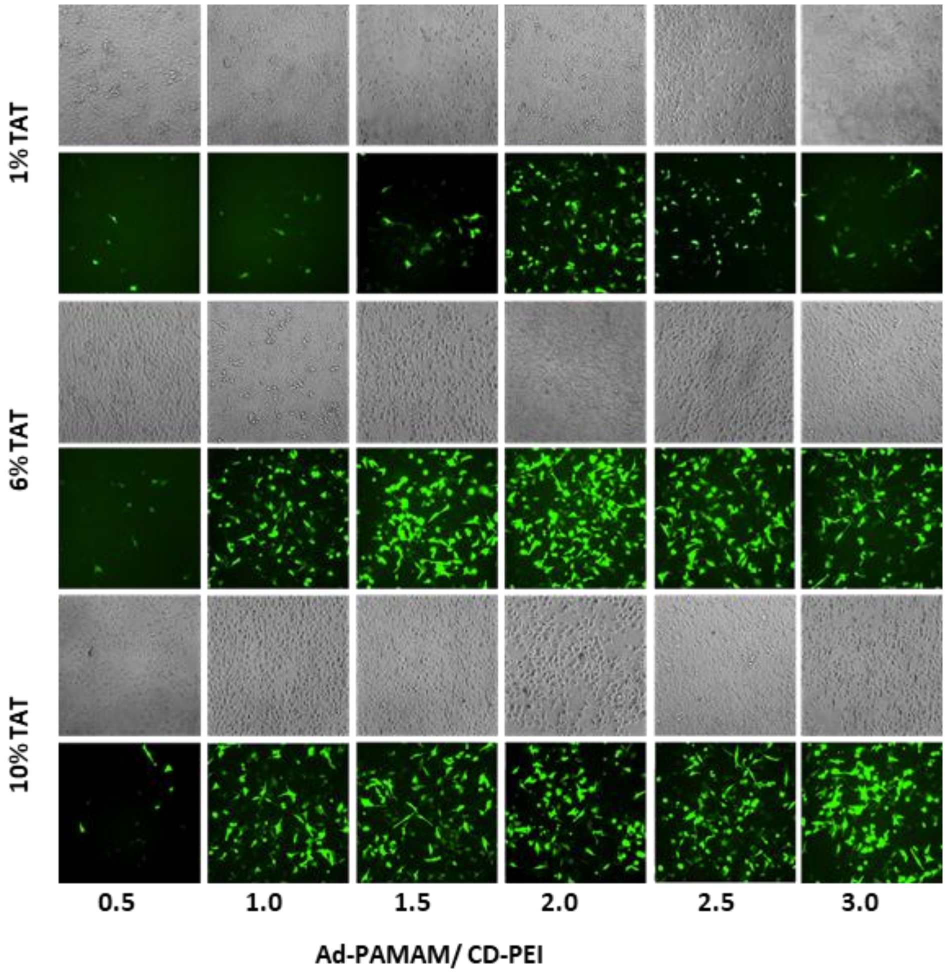

Figure S4. The enlarged fluorescence microscopy images of B16 cells treated by 18 formulations of Donor-RS1/GFP-plasmid⊂SMNPs 48 h post treatment.

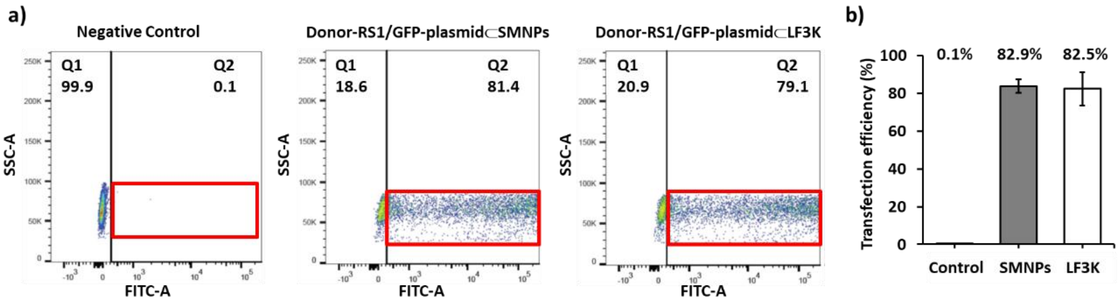

Figure S5. a) Flow-cytometry quantitative analysis of transfection efficiency of the B16 cells at 48 h post treatment with Donor-RS1/GFP-plasmid⊂SMNP or Donor-RS1/GFP-plasmid⊂LF3K. b) Histogram of transfection efficiency of the B16 cells at 48 h post treatment with Donor-RS1/GFP-plasmid⊂SMNP or Donor-RS1/GFP-plasmid⊂LF3K.

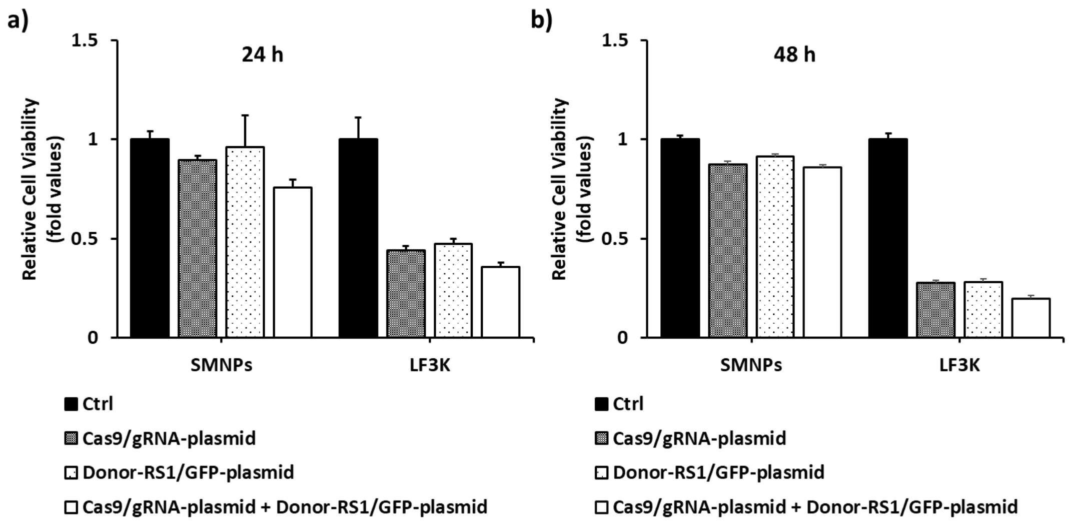

Figure S6. Viability (measured by CCK-8 assay) of B16 cells after treatment with different SMNP vectors and LF3K vectors for (a) 24 h and (b) 48 h.

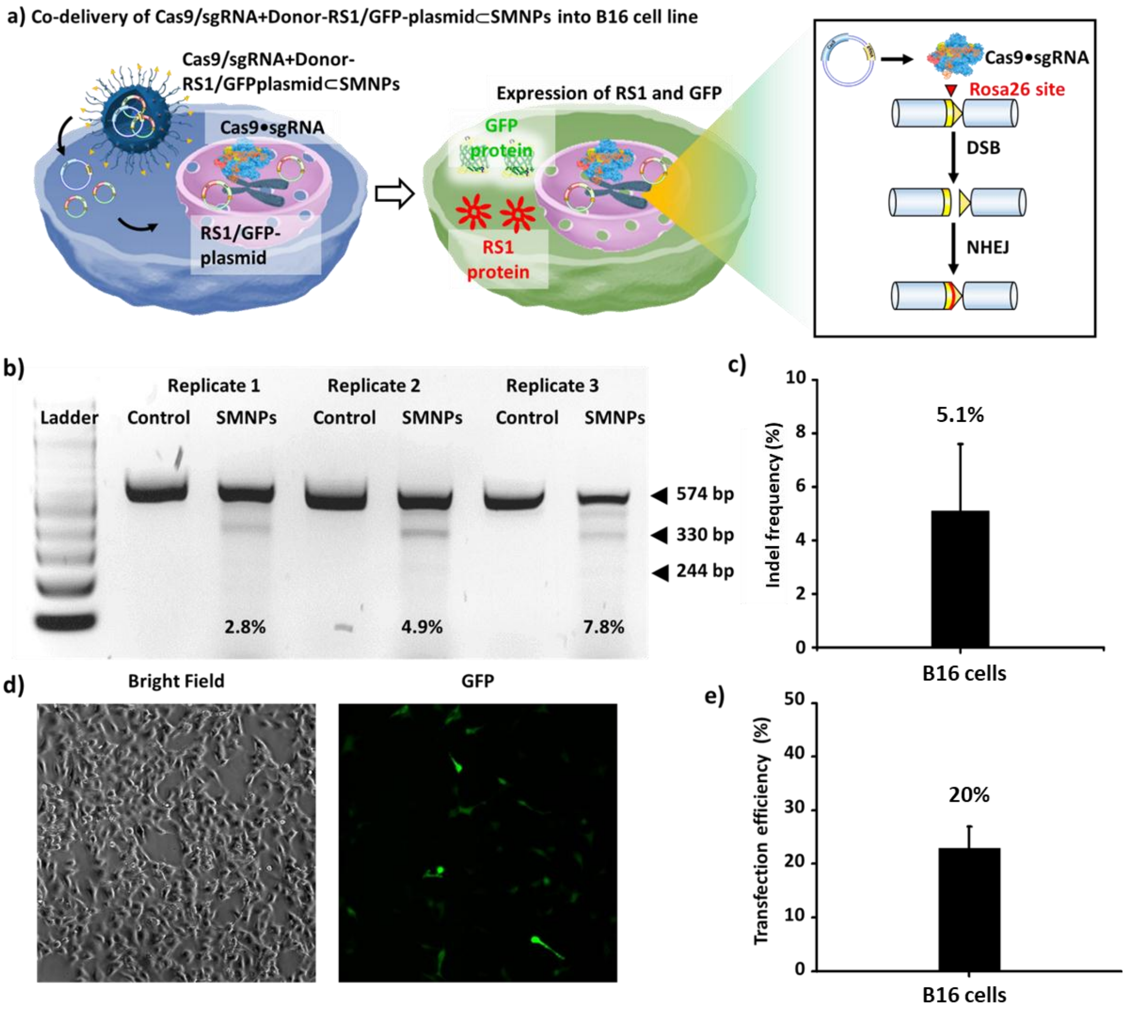

Figure S7. Co-delivery of Cas9/sgRNA-plasmid+Donor-RS1/GFP-plasmid⊂SMNPs into B16 cells. a) Illustration of co-delivery of co-delivery of Cas9/sgRNA-plasmid + Donor-RS1/GFP-plasmid⊂SMNPs into B16 cell line, following CRISPR/Cas9-mediated disruption at the Rosa26 safe-harbor site and GFP expression. b) Electrophoresis was used to quantify the CRISPR/Cas9-mediated disruption performances of the formulation of Cas9/sgRNA-plasmid+Donor-RS1/GFP-plasmid⊂SMNPs. c) Quantitative analysis of the indel frequency in S6c. d) Fluorescence image of cells 2 days post treatment by Cas9/sgRNA-plasmid+Donor-RS1/GFP-plasmid⊂SMNPs. e) Quantitative analysis of the fluorescent micrographs in S6d.

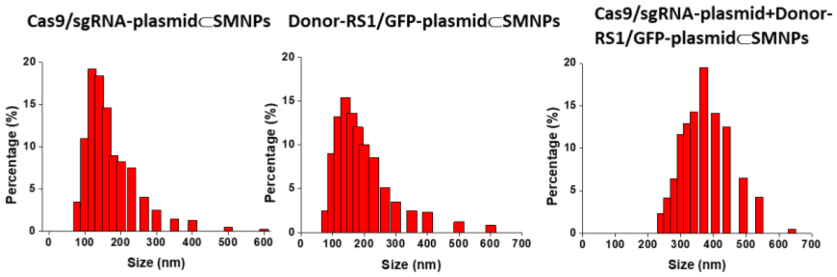

Figure S8. Hydrodynamic sizes of Cas9/sgRNA-plasmid⊂SMNPs, Donor-RS1/GFP-plasmid⊂SMNPs, and Cas9/sgRNA-plasmid+Donor-RS1/GFP-plasmid⊂SMNPs in aqueous media using dynamic light scattering.

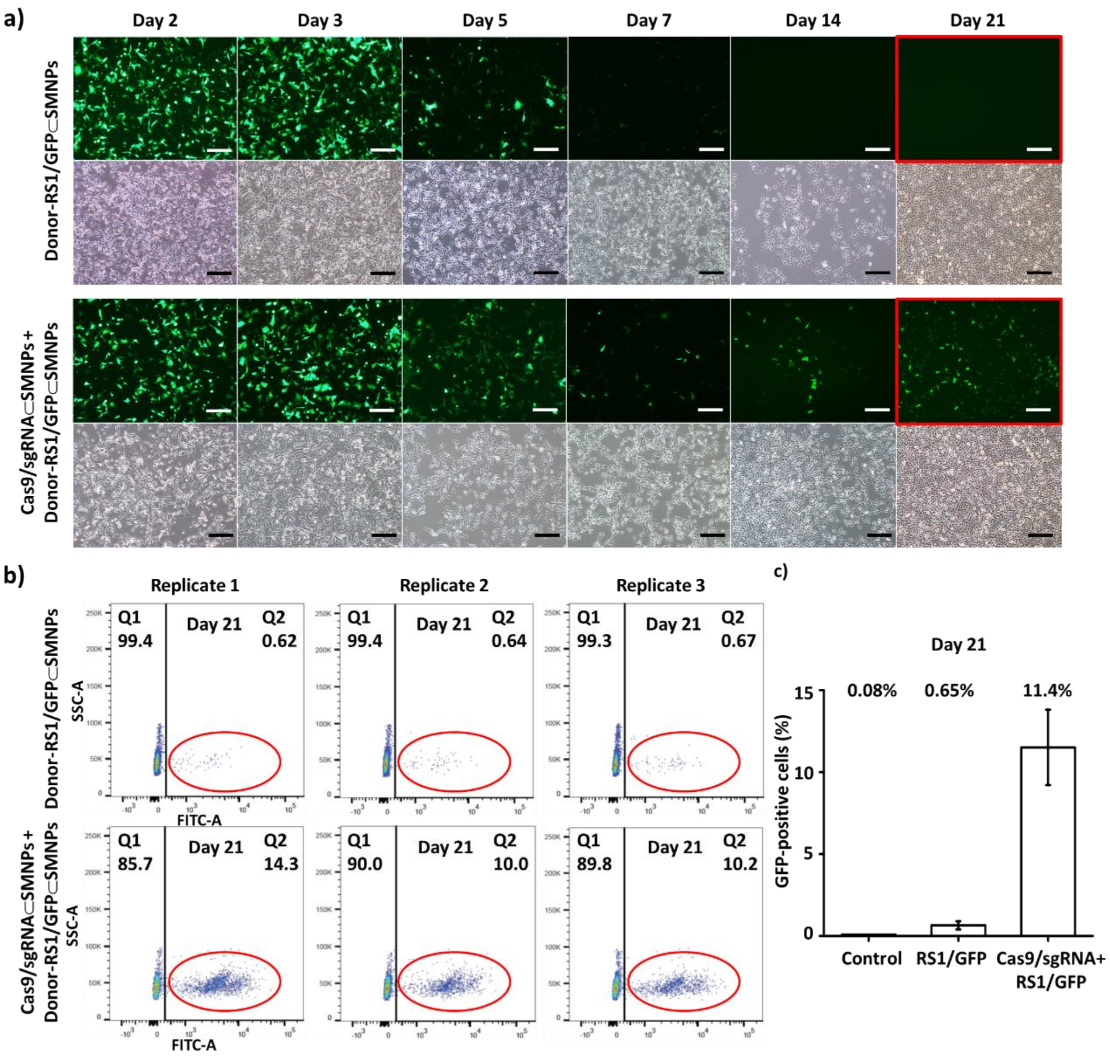

Figure S9. a) Representative fluorescence micrographs of B16 cells treated by Donor-RS1/GFP-plasmid⊂SMNPs alone or both Cas9/sgRNA-plasmid⊂SMNPs and Donor-RS1/GFP-plasmid⊂SMNPs at 2, 3, 5, 7, 14, and 21 days post treatment. Scale bar=200 µm. b) Flow cytometry analysis showing the numbers and ratio of GFP positive cells at day 21. c) Quantitative analysis of the GFP-positive cells via flow-cytometry analysis at day 21. Compared the percentages of GFP-positive cells between Donor-RS1/GFP-plasmid⊂SMNPs alone group (0.65%) and both Cas9/sgRNA-plasmid⊂SMNPs and Donor-RS1/GFP-plasmid⊂SMNPs group (11.4%), the mean knockin efficiency was determined 10.7% (i.e., 11.4% - 0.65% = 10.7%).

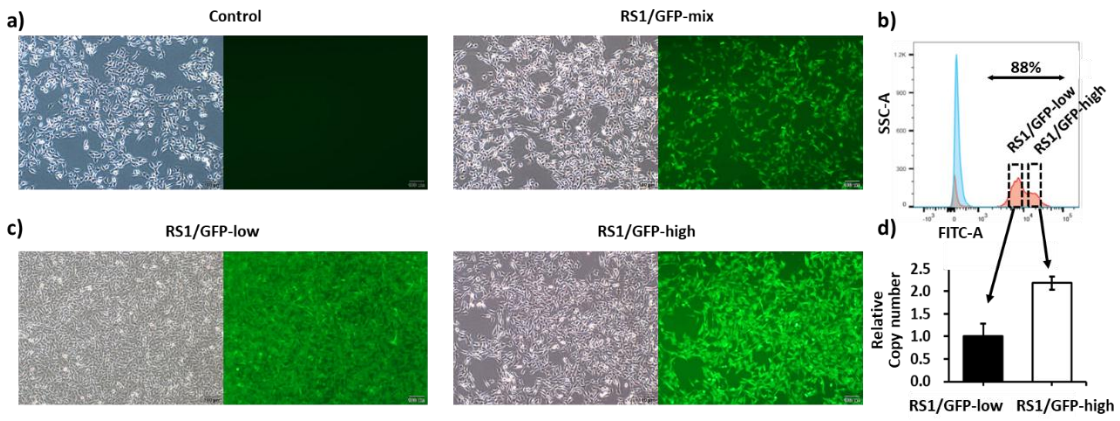

Figure S10. a) Bright-field and fluorescence micrographs images of unedited B16 cells (Control) and sorted RS1/GFP-knockin B16 cells (RS1/GFP-mix). b) Flow cytometry analysis of sorted RS1/GFP-knockin B16 cells, showing two GFP peaks (i.e., RS1/GFP-low and RS1/GFP-high). We further separated the cells via flow cytometry and got RS1/GFP-low B16 cells and RS1/GFP-high B16 cells. c) Bright-field and fluorescence micrographs images of RS1/GFP-low B16 cells and RS1/GFP-high B16 cells. d) The relative copy numbers of RS1/GFP-gene knockin into genomic DNA checked via quantitative PCR. The results indicate that the relative copy numbers of RS1/GFP-gene knockin are 1 for RS1/GFP-low cells (one allele) and 2 for RS1/GFP-high cells (two alleles).

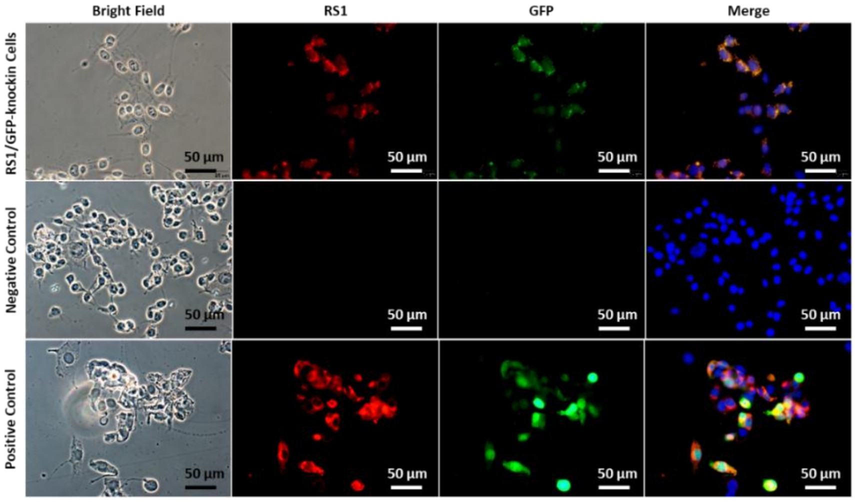

Figure S11. Representative immunofluorescence images of RS1/GFP-knockin B16 cells, a negative control (untreated B16 cells), and a positive control (B16 cells transfected by Donor-RS1/GFP-plasmid at day 2).

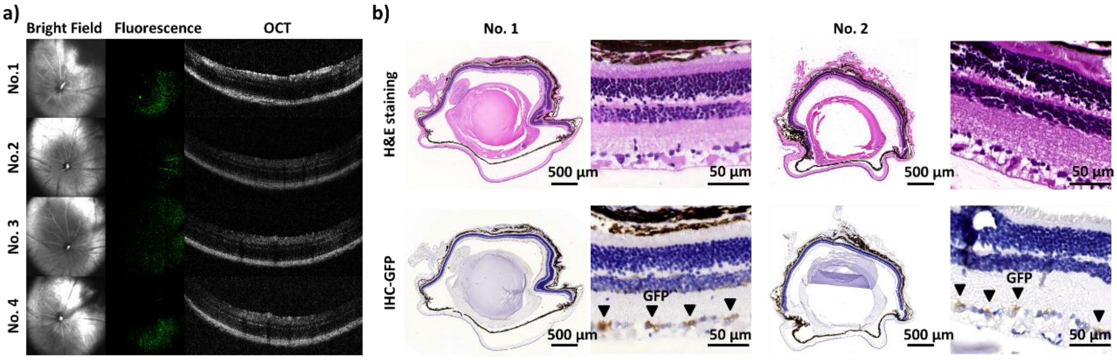

Figure S12. a) A fundus camera and optical coherence tomography (OCT) were employed to detect the GFP signals on retinal surfaces and monitor the anatomical structures of the retinas. b) H&E staining and IHC staining for GFP of the GFP-positive retina tissues.

Reference

[S1] H. Wang, S. Wang, H. Su, K. J. Chen, A. L. Armijo, W. Y. Lin, Y. Wang, J. Sun, K. i. Kamei, J. Czernin, *Angew. Chem. Int. Ed.* **2009**, 48, 4344.

[S2] S. E. Golding, E. Rosenberg, A. Khalil, A. McEwen, M. Holmes, S. Neill, L. F. Povirk, K. Valerie, *J. Biol. Chem.* **2004**, 279, 15402.

[S3] S. Bae, J. Park, J.-S. Kim, *Bioinformatics* **2014**, 30, 1473.
